# A newly developed and validated LC–MS/MS method for measuring 7-dehydrocholesterol (7DHC) concentration in human skin: a tool for vitamin D photobiology research

**DOI:** 10.1007/s43630-022-00274-4

**Published:** 2022-07-29

**Authors:** Oktawia Borecka, Lesley E. Rhodes, Ann R. Webb, John J. Dutton, William D. Fraser

**Affiliations:** 1 Department of Earth and Environmental Sciences, Faculty of Science and Engineering, University of Manchester, Manchester, M13 9PL UK; 2 Division of Musculoskeletal and Dermatological Sciences, Faculty of Medicine Biology and Health, School of Biological Sciences, University of Manchester, Manchester, M13 9PL UK; 3 Photobiology Unit, Dermatology Research Centre, Salford Royal NHS Foundation Trust, Manchester Academic Health Science Centre, Manchester, M6 8HD UK; 4 Bioanalytical Facility, Norwich Medical School, University of East Anglia, Norwich Research Park, Norwich, NR4 7TJ UK; 5 Departments of Clinical Biochemistry and Endocrinology, Norfolk and Norwich University Hospital, Norwich, NR4 7UY UK

**Keywords:** 7-Dehydrocholesterol, Photobiology, Skin, Vitamin D, High-performance liquid chromatography (HPLC), Tandem mass spectrometry (MS/MS)

## Abstract

**Background:**

UVB absorption by 7-dehydrocholesterol (7DHC) in the skin triggers the production of vitamin D and its metabolites, which maintain calcium homeostasis. Detection and measurement of 7DHC in skin using modern liquid chromatography–tandem mass spectrometry (LC–MS/MS) techniques have been lacking, yet there is need for such a technique to provide more information on 7DHC concentration and its UVB responses in human skin.

**Objectives:**

To develop and validate a reliable method to measure 7DHC concentration in skin.

**Methods:**

Human skin punch biopsies of 5 mm diameter obtained through the Manchester Skin Health Biobank were utilised. 7DHC was extracted with ethyl acetate:methanol 1:1 (v/v) and derivatised using 4-phenyl-1,2,4-triazoline-3,5-dione (PTAD), to allow for improved ionisation of 7DHC through Electrospray Ionisation Mass Spectrometry (ESI–MS). Solid supported liquid extraction (SLE) was also employed to allow the removal of larger lipids from 7DHC and minimise potential matrix effects.

**Results:**

The LC–MS/MS assay satisfied International Council for Harmonisation research standards for method validation. Calibration curve was linear with a typical *r*^2^ of 0.997, coefficient of variation was 11.1% and 4.32% for inter-assay and intra-assay imprecision, respectively. Lower limit of quantification was 1.6 µg/g and upper limit of quantification was 100 µg/g, SLE recovery of 7DHC was on average 91.4%.

**Conclusions:**

We have developed a robust, precise and accurate assay for the detection and quantification of 7DHC in small samples of human skin (0.2 cm^2^ surface area). This novel method of extraction and quantification will be valuable to future vitamin D photobiology research.

**Graphical Abstract:**

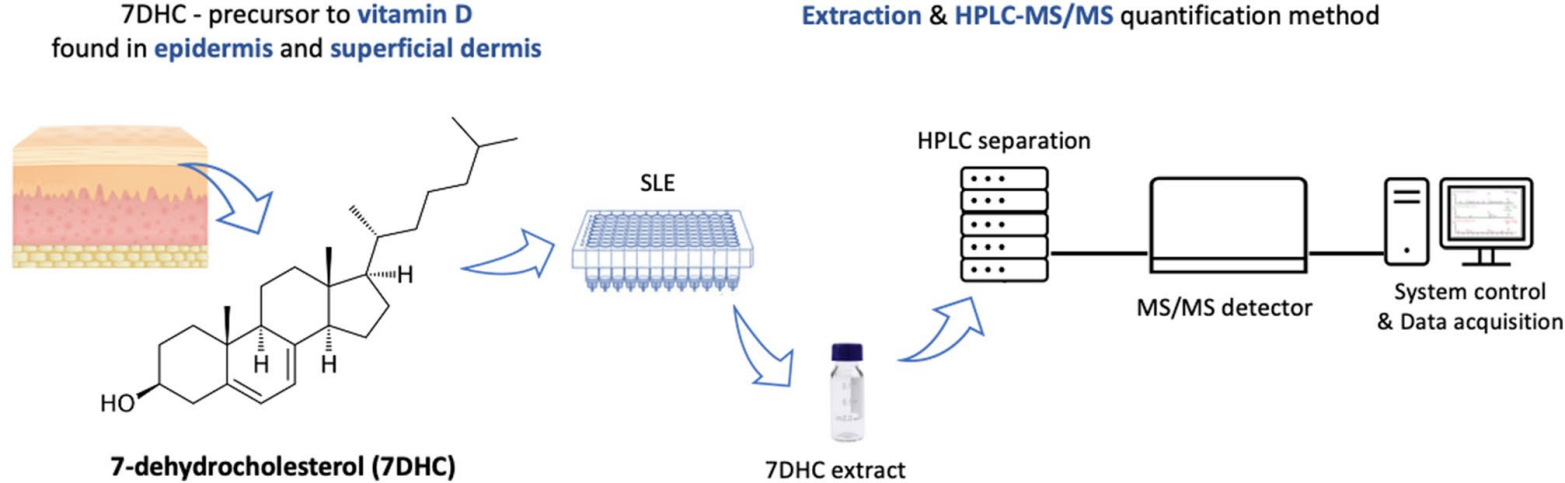

## Introduction

Vitamin D nutrition is important in view of the essential nature of this hormone in musculoskeletal health; it also has roles in immune function and properties that may protect against types of cancer and infection [1, 2]. Vitamin D (cholecalciferol and ergocalciferol) is present in low amounts in the diets of many populations, and there is good evidence that a high proportion of the body’s vitamin D (cholecalciferol) is sourced from the triggering of its synthesis following UVB exposure of precursor 7-dehydrocholesterol (7DHC) in the skin [2–5]. Furthermore, 7DHC is also a substrate for CYP11A1 enzyme in skin and other tissues [6] and can metabolise 7DHC to 7-dehydropregnenolone (7DHP) which has biological activity and can inhibit keratinocyte proliferation [7].

Skin’s 7DHC is reported to be more abundant in the upper, epidermal layer than the lower, dermal layer [8]. As it is the precursor required for vitamin D synthesis by the skin, there is considerable interest in an assay measuring concentration of 7DHC in skin accurately, particularly in human in vivo studies. There are 7DHC assay methods described in the literature [9–14], capable of detecting 7DHC in various tissues, such as blood, plasma, amniotic fluid, cultured skin fibroblasts and neuroblastoma cell lines. However, these types of matrices are easier to extract from and avoid matrix effects, compared to skin which is more difficult to work with due to the prevalence of lipids and collagen fibres. Despite advances in quantitative analytical techniques for 7DHC concentration measurements, there are, to our knowledge, no recent developments in assays making them capable of robust 7DHC measurement in small samples of human skin, suitable for in vivo studies, and in particular employing tandem mass spectrometry, which is considered the ‘gold standard’ for the quantitative measurement of vitamin D metabolites in serum due to its sensitivity, specificity and reliability [15].

Previous skin 7DHC assays relied on chromatographic separation, while using a UV detector [8, 16–18], which is known to provide challenges, since it is able to indicate different retention times of molecules, but not able to distinguish between different molecular weights, hence potentially producing less reliable results. These previous assays were not, by modern standards, fully validated. No other studies of 7DHC concentration in skin used a MS/MS detection system [19].

7DHC and other vitamin D derivatives are known to be poorly ionisable compounds [20]. Since ionisation is a key factor for ESI–MS detection, it is beneficial to use derivatising agents to enhance the detection of 7DHC [12, 21, 22]. Commercially available 4-phenyl-1,2,4-triazoline-3,5-dione (PTAD) in a Diels–Alder cycloaddition reaction has been used as a basis for many vitamin D metabolite assays in the past, and yielded excellent results [23, 24]. Based on this knowledge and technology, we aimed to develop and validate a reliable method to measure 7DHC concentration in skin.

## Materials and methods

### Skin sample collection

Skin samples used for the development of the method were obtained through Manchester Skin Health Biobank (MSHBiobank) from healthy adults undergoing liposculpture procedures. The skin samples were collected using a 5 mm diameter hollow, circular scalpel (punch biopsy instrument) from the piece of skin provided. The study was approved by the North West Research Ethics Committee (reference 14/NW/0185). All patients gave written, informed consent. Porcine skin samples obtained fresh from a local butcher shop were also used in early stages of the development of the assay.

### Materials, calibration standards and controls

7DHC (≥ 95% chemical purity) and 4-phenyl-1,2,4-triazoline-3,5-dione (PTAD; 97% chemical purity) were purchased from Sigma-Aldrich Chemicals Company Ltd, Dorset, UK. Stable isotope labelled 3β-7-DehydroCholesterol-d7 (3β-7DHC-d7; 98% chemical purity) was purchased from Toronto Research Chemicals, Toronto, Canada. 7DHC, 3β-7DHC-d7 and PTAD were stored at − 20 °C in sealed amber glass ampoules prior to use, to avoid loss of UVR spectral integrity (due to photoconversion). Deionised water (H_2_O), LCMS grade methanol (MeOH) (≥ 99.9%) and acetonitrile (ACN) (≥ 99.9%), analytical reagent grade ethyl acetate (EA) (≥ 99%) and propan-2-ol and HPLC grade hexane and formic acid were purchased from Fisher Scientific, Loughborough, UK.

Stock solutions, containing 20 µg/mL 7DHC in ACN and 5 µg/mL 7DHC Internal Standard (IS) in MeOH or ACN, were prepared and stored at + 4 °C. 7DHC stock solution was then further diluted with ACN to obtain working concentrations as outlined in the sample preparation procedure. New stock solutions were prepared every 2–3 weeks, as there was a loss of signal in solutions older than 2 months.

### Sample preparation procedure for LC–MS/MS

Subcutaneous fat was removed from 5 mm human skin punch biopsies (0.2 cm^2^ surface area) using a surgical scalpel. Aqueous and skin samples were then processed in the same manner. 50 µL of 5 µg/mL 7DHC IS was added alongside 5 mL of EA:MeOH 1:1 (v/v) to each sample. Samples were rotor mixed for 2 min at 40 revolutions per minute (rpm), placed in a sonicator for 30 min then centrifuged at 4000 rpm for 10 min. Supernatant (1 mL) was transferred to borosilicate tubes and dried at 60 °C under a constant stream of nitrogen. 100 µL of propan-2-ol was added and the test tube was vortexed for 30 s. Next, 200 µL of water was added and vortexed again for 30 s. The samples were then transferred to a Solid supported liquid extraction (SLE) plate, and after 5 min, 750 µL of hexane was added to each well. After a further 5 min, an additional 750 µL of hexane was added and left for 5 min, after which time a vacuum was applied to draw through any residual hexane fraction. The liquid extracted through SLE was then dried under a constant stream of nitrogen at 60 °C. 1 mL of PTAD in ACN (100 mg per 500 mL) was then added, rotor mixed and allowed to stand for 30 min at room temperature, for the derivatisation reaction to take place. After 30 min, the reaction was stopped with addition of 750 µL of water and each sample in the plate was mixed using a separate pipette. The plate was finally manually shaken for 2 min and placed in the auto-sampler for LC–MS/MS analysis.

### Liquid chromatography

Extracted sample introduction and chromatography were achieved with a CTC Analytics HTC PAL autosampler (CTC Analytics, Zwingen, Switzerland), fitted with a chill stack maintained at 10 °C ± 2 °C. An Agilent 1100 series high-performance liquid chromatography (HPLC) system (Agilent Technologies, Cheadle, UK), consisting of binary pump, solvent degasser and column oven (maintained at 40 °C). 50 µL was used as the injection volume to the system, fitted with a 30 µL loop using total loop overfill mode. Chromatographic separation was achieved using a 2.7 µm Modus pentafluorophenyl (PFP) 100 mm × 2.1 mm reversed-phase column (Chromatography Direct, Runcorn, UK). Chromatographic separation of the PTAD derivatised 7DHC was performed using a gradient elution profile of 65% Solvent B, 35% Solvent A at 0 and 2 min; 95% Solvent B and 5% Solvent A at 3 and 5 min; 65% Solvent B and 35% Solvent A at 7 min. Flow rate was 0.4 mL/min throughout. Mobile phase A consisted of 0.1% Formic Acid in LCMS Grade Water and mobile phase B was 0.1% Formic Acid in LCMS Grade ACN. Total run time was 14 min.

### Tandem mass spectrophotometry analysis

**Fig. 1:**
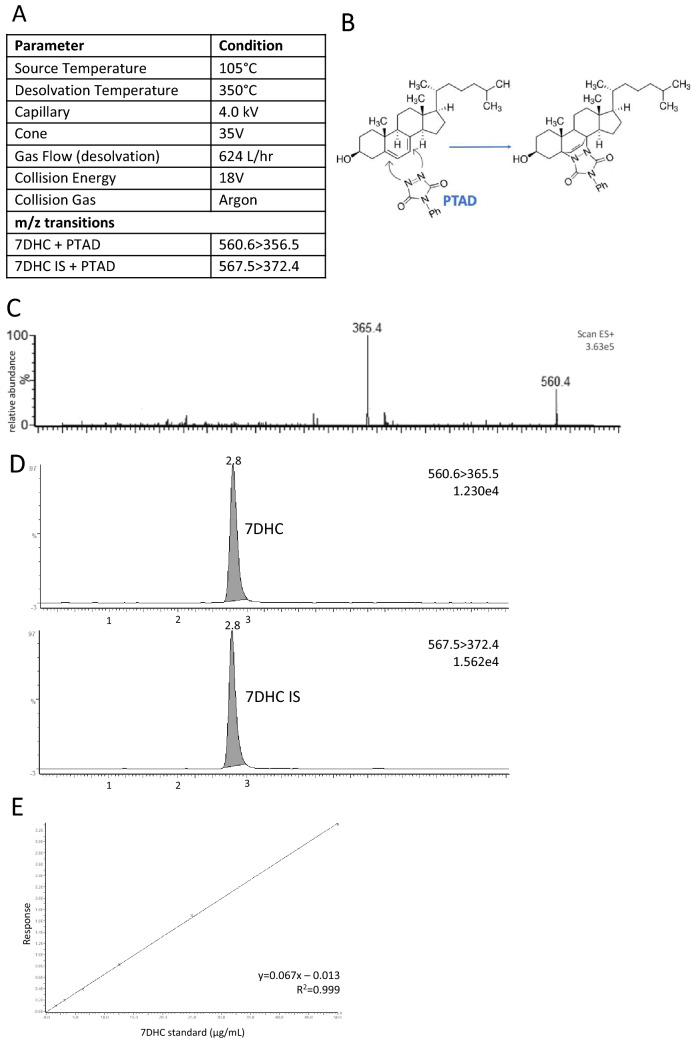
**A** Mass spectrometer parameter settings and MRM precursor to product ion transitions for 7DHC/PTAD and 7DHC IS/PTAD. **B** Diels–Alder reaction of 7DHC with PTAD and formation of one of the adducts. **C** Collision-induced dissociation spectra of PTAD-derivatized 7DHC. The precursor to product ion transition *m*/*z* 560.6 > 356.5 was utilised for MRM. **D** Chromatogram from the standard containing 12.5 µg/mL of 7DHC and 5 µg/mL IS 7DHC. **E** Typical standard curve constructed by plotting the relative response of each standard on the *y*-axis against their respective concentrations (µg/mL) on the *x*-axis. Regression analysis presented a typical correlation coefficient *r*^2^ > 0.99

Analyses were performed on a MicroMass Ultima Pt mass spectrometer (Waters Corp., Milford, MA, USA). MS/MS detection was performed using ESI in the positive ion mode, data was acquired using Waters MassLynx 4.1 software package (Waters Corp., Milford, MA, USA). System control, data acquisition, baseline integration and peak quantification was achieved using Waters QuanLynx software. The optimisation of MS/MS parameters was performed by direct infusion of derivatised standard (see Fig. 1A).

### Method validation

**Fig. 2:**
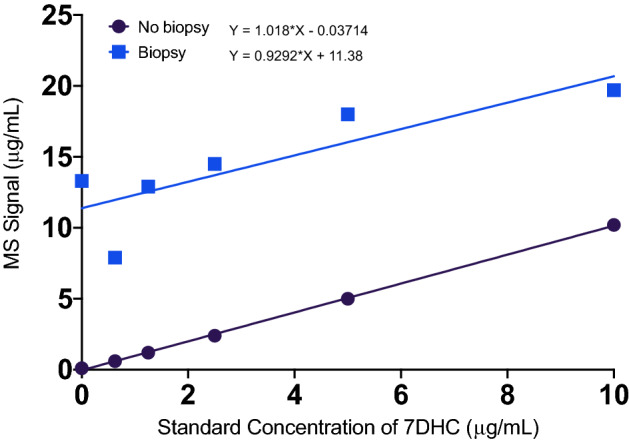
Matrix effect experiment. MS signal vs concentration of 7DHC obtained by spiking aqueous solutions and human skin biopsy samples with the known concentration of 7DHC standard

Certified pure standards of 7DHC and 3β-7DHC-d7 (Toronto Research Chemicals, Canada) were used and spiked gravimetrically into either aqueous solutions or sample skin biopsies representing the same biological matrix as the samples. The comparison of slopes was made on six spiked aqueous solutions and six skin biopsy samples spiked with the same concentrations of 7DHC and 7DHC IS (Fig. 2).

### Linearity

**Fig. 3:**
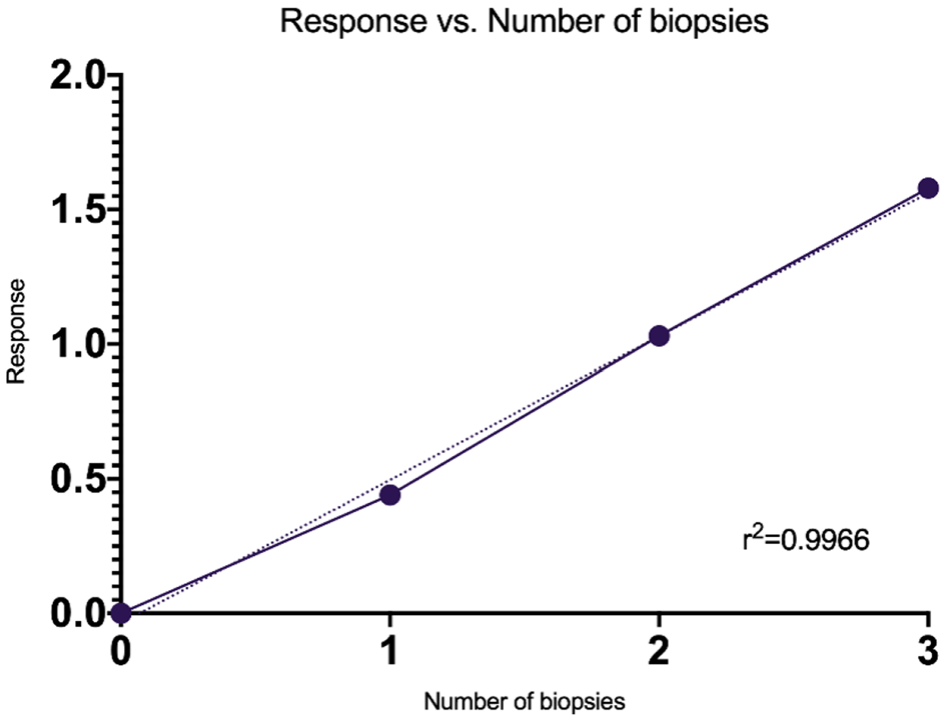
Response vs. the number of porcine skin biopsies used. Dotted line represents the line of best fit

7DHC standards with concentrations of 1.6, 3.13, 6.25, 12.5, 25, 50 µg/mL were used to generate calibration curves. A standard curve was generated by plotting the ratio of analyte peak area to internal standard peak area on the *y*-axis against the weighted (1/*x*) concentration of their respective standards on the *x*-axis. *R*-squared was used as goodness-of-fit measure for linear regression model; correlation coefficient (*r*^2^) values of > 0.95 were accepted. Linearity was also assessed using multiples of porcine skin biopsies, to assess if the peak area of 7DHC response is increasing with increasing number of the biopsies (Fig. 3).

The data in Fig. 3 shows that the peak area of 7DHC is increasing with increasing number of biopsies, while the internal standard is compensating for any loss during the extraction procedure. This suggests that the extraction procedure was efficient and there is a linear correlation between the amount of tissue used and the peak area of the curves.

### Accuracy, precision and recovery

As the accuracy of an analytical method could be influenced by sample matrix and lead to ion suppression, where possible, the analytical method was assessed using human skin biopsies, where it was not possible porcine skin or aqueous solutions were used. The spike experiment was carried out on 6 spiked human skin biopsies and 6 spiked solutions not containing biopsies across the analytical range established in earlier experiments to test the effects of the biological matrix on the efficiency of the extraction (Fig. 2).

Imprecision of the assay was assessed by six consecutive measurements of calibration curves for in-batch (intra-assay) and between-batch (inter-assay) variability. Precision is expressed as coefficient of variation (CV), our acceptance criteria defined the intra-assay CV limit of < 10% at one concentration and cumulative inter-assay CV limit < 15% across the assay. Both standard deviation and coefficient of variation (CV) were calculated and used to determine the imprecision of the method.

### Lower limit of quantification and detection

A precision profile was also carried out. Lower limit of quantification (LLoQ) and detection (LLoD) of the assay were determined. Standards containing 7DHC at concentration of 0.024, 0.048, 0.1, 0.2, 0.39, 0.78, 1.6, 3.13, 6.25, 12.5, 25 and 50 µg/mL were analysed three times. The CV of each sample was plotted against their respective concentration. Signal-to-noise (s/n) ratio has to be 10:1 for the analyte peak to establish LLoD and the LLoQ was defined as the concentration at which the CV ≤ 22%.

## Results

Chromatographic peak of 7DHC was eluted at 2.8 min (Fig. 1D). Total injection-to-injection run time was 14 min. The slopes in the matrix effects experiment of the aqueous solutions and spiked biopsies suggest no ion suppression (Fig. 2). Based on chromatograms and MS data the method developed did not result in the presence of significant quantifiable oxidised forms of 7-DHC.

### Method validation, recovery efficiency and removal of phospholipids

The assay validation is summarised in Table 1. The assay achieved adequate linearity between 0 and 100 µg/mL. The assay demonstrated sensitivity, precision and satisfactory recovery. Injections of aqueous blanks after running standard 12.5 µg/mL 7DHC spiked samples confirmed the absence of carry-over of the analyte.

**Table 1:** HPLC–MS/MS assay characteristics

	7DHC
Linearity, µg/mL	0–100
Typical *r*^2^	0.997
Intra-assay imprecision; mean %CV (at 12.5 µg/mL)	4.32%
Inter-assay imprecision; mean %CV (range: 1.6–50 µg/mL)	11.1%
LLoQ, µg/g	1.6
ULoQ, µg/g	100^a^
Mean SLE recovery of 7DHC concentration	91.4% (*n* = 6)

^a^The upper limit of quantification (ULoQ) was established based on our method using 1 mL of PTAD. The competition for PTAD between 7DHC IS and increasing concentrations of 7DHC led to 7DHC IS area decreasing with higher 7DHC concentrations. The cut off of 100 µg/g was established by calculating 7DHC IS percentage difference from the first three samples with lower 7DHC concentration and their 7DHC IS area. The percentage difference of > 20% was used as a cut off

An experiment was carried out on a single human skin biopsy (Table 2). The same biopsy was re-extracted four times (E1–E4) with diminishing extraction of material each time. The result of the experiment gave 96% 7DHC recovery on the first tissue extraction procedure, then more extractions of the same biopsy followed. 96% extraction efficiency was calculated by taking 7DHC area of the 1st extraction and dividing it by sum of all four 7DHC extractions, then multiplied by 100% [6018/(6018 + 220 + 19 + 3) * 100% = 96%]. The first tissue extraction procedure 7DHC IS recovery was 97%. This was calculated in the same way as described above [58171/(58,171 + 1763 + 86 + 12) * 100% = 97%].

**Table 2:** Recovery experiment of human skin biopsy experiment

	RT	RT IS	Area	IS area	Response	S/N
E1	2.87	2.86	6018	58,171	0.10	2004
E2	2.83	2.82	220	1763	0.12	684
E3	2.83	2.82	19	86	0.22	53
E4	2.93	2.84	3	12	0.30	16

7DHC area and 7DHC IS area signals decrease after each time extraction process is carried out on the same skin biopsy

*E* extraction, *RT* retention time, *IS* internal standard, *S/N* signal to noise

## Discussion

We describe the validation of an LC–MS/MS assay for the measurement of 7DHC in small samples of human skin. The inclusion of SLE allowed effective removal of potentially interfering phospholipids and ceramides found in skin. The method demonstrated a mean 91.4% recovery of 7DHC using SLE.

Derivatisation with PTAD was an essential step to allow better ionisation of 7DHC, by adding oxygen and nitrogen molecules which are easily ionised. Shifting the *m*/*z* ratio from 384.6 to 559.6 through derivatization, enabled better separation from lower molecular weight species while also making the analyte more hydrophobic, hence diluting later than the native molecules. The working range of the assay (1.6–100 µg/mL) has been established based on sample skin biopsies, with ULoQ of 100 µg/g and LLoQ of 1.6 µg/g sufficient to detect subjects with a low concentration of 7DHC in their skin.

Early, labour and material intensive (*L*: 10 cm × *W*: 2 cm) attempts using sterol chromatography and UV spectroscopy aimed to quantify the composition of human epidermal lipids. 7DHC was identified using its UV absorption spectrum with characteristic peaks at 260, 270, 281, and 293 nm [16] and assessed to be on average 4% of the total sterols found in epidermis. This value was later argued to be considerably lower [19]. The assay used was not fully validated. Ten years later a research group from Texas Research Institute separated vitamin D and its metabolites in human skin, including 7DHC, using gas chromatography; however, little quantification was involved [17].

In the 1980s Holick and his colleagues developed a new method for the quantification of 7DHC and pre-vitamin D_3_ in skin. It employed high-performance liquid chromatography (HPLC) with UV detection at 254 nm [8]. As stable isotopically labelled internal standards of 7DHC were not commercially available, 7DHC isotopically labelled with tritium (^3^H) was produced “in-house” with previously described methods [25, 26], while large 6.25 cm^2^ surface area samples of skin tissue were used for the analysis. This method was later used as the basis for an experiment in the 1980s, which examined 7DHC concentration differences between skin of young and older adults [18].

A group from Dundee Hospital Medical School, Scotland, developed an improved method for the measurement of 7DHC in skin [19], with the aim to increase the sensitivity of 7DHC measurements. These authors suggested that a single-stage isocratic HPLC procedure does not provide sufficient sensitivity; therefore, they supplemented their HPLC–UV system with an amperometric detector, which allowed them to use smaller skin samples, with better separation of analyte and its internal standard. The samples they used were significantly smaller (5 mm × 5 mm/0.25 cm^2^) than in earlier experiments. They were also able to purchase industrial grade 7DHC, cholesterol and ergosterol (used as internal standard) needed for analyses. The method was validated for 7DHC recovery after the addition of 7DHC to skin homogenates and demonstrated absolute recoveries ranging from 30 to 75%; intra-batch and inter-batch imprecision was also evaluated and was 2.1% (20 µg/mL) and 4.6–12.9% (4, 8, 12, 16 and 20 µg/mL), respectively.

While the Dundee Hospital Medical School group assay [19], appeared the most reliable technique for the assessment of 7DHC concentration in human skin, advances in analytical techniques can aid the development of a more reliable method. With the increasing use of MS rather than UV detection and the availability of commercially available standards for 7DHC and stable isotopically labelled 7DHC internal standards, refinement of the 7DHC quantification technique can be achieved. A more complete validation of the 7DHC assay, compared to previous methods, assures that the new technique is reproducible and obtains good 7DHC recovery from skin.

The previous experiments aiming to quantify 7DHC (and pre-vitamin D_3_) in skin using a UV detector emphasised the limitations of these methods [8, 16, 17, 19]. While a UV detector is able to indicate different retention times, it is not able to distinguish between different molecular weights. This raises concern that other compounds with similar retention time to 7DHC are being detected in the 7DHC peaks produced using a UV detector. Hence, interpreting UV chromatograms can be extremely challenging. Due to the use of MS/MS we can be confident that compounds with similar retention time are not interfering with the measurement of 7DHC.

LC–MS/MS system also allows for higher sensitivity and use of smaller skin samples (0.2 cm^2^ surface area). This is particularly important when collecting samples from human volunteers, rather than skin as a waste product from various surgical procedures. The use of punch biopsies from healthy volunteers allows for better understanding of processes taking place in healthy, ‘alive’ skin and it is a generally used and minimally invasive procedure in dermatology research [27–29]. Compared to previous studies, we developed a significantly more sensitive assay requiring much less tissue.

We also applied standard validation procedures to our assay, which were mostly omitted in previous studies; and carried out matrix effects experiment with human skin biopsies (Fig. 2), not performed in previous assay evaluations. This allowed us to see that overall, 7DHC spiked human samples and aqueous samples behave in a similar manner, and we were not experiencing any major matrix effects.

In conclusion, the use of LC–UV spectrophotometry brings about some issues which can be addressed with the use of LC–MS/MS. The biggest shortcoming of using a UV detector is its inability to distinguish between different molecular weights, in the way that MS does. We believe our validated method is a significant improvement in the measurement of 7DHC in human skin.

This new method brings many opportunities for application in photobiology research. This includes skin 7DHC response to UVB exposure in different age groups, in normally unexposed vs regularly sun-exposed body sites, and the influence of skin disease, such as psoriasis or vitiligo. For example, future research could revisit age-related ability to synthesise vitamin D from 7DHC upon UVB exposure, with protocols involving well-matched volunteers of different age groups and assessment of matched skin sites, or explore responses of skin 7DHC to physiological doses of solar simulated radiation. This would provide better understanding of the reasons for differences in vitamin D status and have public health significance in view of the importance of vitamin D nutrition [1, 2], including in the context of the growing 65+-year-old population in many countries [30, 31].

## References

[1] SACN. (2016). Vitamin D and health. Retrieved January 28, 2020, from https://www.gov.uk/government/uploads/system/uploads/attachment_data/file/537616/SACN_Vitamin_D_and_Health_report.pdf.

[2] AGNIR. (2017). Ultraviolet radiation, vitamin D and health: report of the independent advisory group on non-ionising radiation.

[3] Webb AR, Kift R, Durkin MT, OBrien SJ, Vail A, Berry JL, Rhodes LE (2010). The role of sunlight exposure in determining the vitamin D status of the U.K. white adult population. British Journal of Dermatology.

[4] Kift R, Berry JL, Vail A, Durkin MT, Rhodes LE, Webb AR (2013). Lifestyle factors including less cutaneous sun exposure contribute to starkly lower vitamin D levels in U.K. South Asians compared with the white population. British Journal of Dermatology.

[5] Rhodes LE, Webb AR, Berry JL, Felton SJ, Marjanovic EJ, Wilkinson JD, Vail A, Kift R (2014). Sunlight exposure behaviour and vitamin D status in photosensitive patients: Longitudinal comparative study with healthy individuals at U.K. latitude. British Journal of Dermatology.

[6] Slominski RM, Raman C, Elmets C, Jetten AM, Slominski AT, Tuckey RC (2021). The significance of CYP11A1 expression in skin physiology and pathology. Molecular and Cellular Endocrinology.

[7] Slominski AT, Kim T-K, Chen J, Nguyen MN, Li W, Yates CR, Sweatman T, Janjetovic Z, Tuckey RC (2012). Cytochrome P450scc-dependent metabolism of 7-dehydrocholesterol in placenta and epidermal keratinocytes. International Journal of Biochemistry & Cell Biology.

[8] Holick M, MacLaughlin J, Clark M, Holick S, Potts J, Anderson R, Blank I, Parrish J, Elias P (1980). Photosynthesis of previtamin D3 in human skin and the physiologic consequences. Science.

[9] Kelley RI (1995). Diagnosis of Smith-Lemli-Opitz syndrome by gas chromatography/mass spectrometry of 7-dehydrocholesterol in plasma, amniotic fluid and cultured skin fibroblasts. Clinica Chimica Acta.

[10] Johnson DW, Brink HJ, Jakobs C (2001). A rapid screening procedure for cholesterol and dehydrocholesterol by electrospray ionization tandem mass spectrometry. Journal of Lipid Research.

[11] Gelzo M, Clericuzio S, Barone R, D’Apolito O, Russo AD, Corso G (2012). A routine method for cholesterol and 7-dehydrocholesterol analysis in dried blood spot by GC-FID to diagnose the Smith-Lemli-Opitz syndrome. Journal of Chromatography B: Analytical Technologies in the Biomedical and Life Sciences.

[12] Liu W, Xu L, Lamberson C, Haas D, Korade Z, Porter NA (2014). A highly sensitive method for analysis of 7-dehydrocholesterol for the study of Smith-Lemli-Opitz syndrome. Journal of Lipid Research.

[13] Becker S, Röhnike S, Empting S, Haas D, Mohnike K, Beblo S, Mütze U, Husain RA, Thiery J, Ceglarek U (2015). LC-MS/MS-based quantification of cholesterol and related metabolites in dried blood for the screening of inborn errors of sterol metabolism. Analytical and Bioanalytical Chemistry.

[14] Xu L, Kliman M, Forsythe JG, Korade Z, Hmelo AB, Porter NA, McLean JA (2015). Profiling and imaging ion mobility-mass spectrometry analysis of cholesterol and 7-dehydrocholesterol in cells via sputtered silver MALDI. Journal of the American Society for Mass Spectrometry.

[15] Fraser WD, Tang JCY, Dutton JJ, Schoenmakers I (2020). Vitamin D measurement, the debates continue, new analytes have emerged, developments have variable outcomes. Calcified Tissue International.

[16] Reinertson RP, Wheatley VR (1959). Studies on the chemical composition of human epidermal lipids. The Journal of Investigative Dermatology.

[17] Rauschkolb EW, Davis HW, Fenimore DC, Black HS, Fabre LF (1969). Identification of vitamin D3 in human skin. The Journal of Investigative Dermatology.

[18] MacLaughlin J, Holick MF (1985). Aging decreases the capacity of human skin to produce vitamin D3. Journal of Clinical Investigation.

[19] Moody JP, Humphries CA, Allan SM, Paterson CR (1990). Determination of 7-dehydrocholesterol in human skin by high performance liquid chromatography. Journal of Chromatography.

[20] Strathmann FG, Laha TJ, Hoofnagle AN (2011). Quantification of 1α,25-dihydroxy vitamin D by immunoextraction and liquid chromatography–tandem mass spectrometry. Clinical Chemistry.

[21] Hedman CJ, Wiebe DA, Dey S, Plath J, Kemnitz JW, Ziegler TE (2014). Development of a sensitive LC/MS/MS method for vitamin D metabolites: 1,25 dihydroxyvitamin D2&3 measurement using a novel derivatization agent. Journal of Chromatography B: Analytical Technologies in the Biomedical and Life Sciences.

[22] Higashi T, Shimada K, Toyo’oka T (2010). Advances in determination of vitamin D related compounds in biological samples using liquid chromatography–mass spectrometry: A review. Journal of Chromatography B: Analytical Technologies in the Biomedical and Life Sciences..

[23] Tang JCY, Jackson S, Walsh NP, Greeves J, Fraser WD, Bioanalytical Facility team (2019). The dynamic relationships between the active and catabolic vitamin D metabolites, their ratios, and associations with PTH. Science and Reports.

[24] Tang JCY, Nicholls H, Piec I, Washbourne CJ, Dutton JJ, Jackson S, Greeves J, Fraser WD (2017). Reference intervals for serum 24,25-dihydroxyvitamin D and the ratio with 25-hydroxyvitamin D established using a newly developed LC–MS/MS method. Journal of Nutritional Biochemistry.

[25] Holick MF, Frommer JE, McNeill SC, Richtand NM, Henley JW, Potts JT (1977). Photometabolism of 7-dehydrocholesterol to previtamin D3 in skin. Biochemical and Biophysical Research Communications.

[26] Jones G, Schnoes HK, DeLuca HF (1975). Isolation and identification of 1,25-dihydroxyvitamin D2. Biochemistry.

[27] Felton SJ, Shih BB, Watson REB, Kift R, Webb AR, Rhodes LE (2020). Photoprotection conferred by low level summer sunlight exposures against pro-inflammatory UVR insult. Photochemical & Photobiological Sciences.

[28] Langton AK, Graham HK, Griffiths CEM, Watson REB (2019). Ageing significantly impacts the biomechanical function and structural composition of skin. Experimental Dermatology.

[29] Shih BB, Farrar MD, Cooke MS, Osman J, Langton AK, Kift R, Webb AR, Berry JL, Watson REB, Vail A, de Gruijl FR, Rhodes LE (2018). Fractional sunburn threshold UVR doses generate equivalent vitamin D and DNA damage in skin types I–VI but with epidermal DNA damage gradient correlated to skin darkness. The Journal of Investigative Dermatology.

[30] Christensen K, Doblhammer G, Rau R, Vaupel JW (2009). Ageing populations: The challenges ahead. Lancet.

[31] Office for National Statistics. (2011). Census: Population estimates for the United Kingdom.

